# Supplementary material for: Resection and repair of a Cas9 double-strand break at CTG trinucleotide repeats induces local and extensive chromosomal deletions
Source: PLoS Genet. 2020 Jul 16;16(7):e1008924. doi: 10.1371/journal.pgen.1008924 (PMC7413560; doi:10.1371/journal.pgen.1008924)
Supplement: S1 Fig — Junctions were deduced from Illumina read mapping (when available) and confirmed by subsequent PCR and Sanger sequencing. Nucleotides in red are those used to anneal each DSB end, and are therefore present in only one copy in the genomic sequence. The extent of calculated deletions (Δ) is indicated in parentheses. Nucleotides in red in parentheses correspond to small deletions. Nucleotides in green correspond to insertions. The length of Ty insertions is indicated along with the LTR it comes from. Nucleotides in purple (SpCas9 at the ARG2 locus) correspond to the I-SceI site (see text). Nucleotides in light blue correspond to homeologies between the left and right junction sequences that were lost after deletion (the junction sequence shows the nucleotide in blue, not the one in red). Nucleotides in light blue in parentheses correspond to homeologies that were removed during the deletion. Note that extended homologies between LTRs does not always allow to determine the exact breakpoint with a high precision. (PDF) [file pgen.1008924.s001.pdf]

Wild-type *Sp*Cas9 junctions (at the *SUP4* locus)

Parental allele	GGAATG CTG$_{90}$ CTGGGGGGATCACAGACCAT
#2	GGAATG CTG$_{21}$ ATGCTGGGGGGATCACAGACCAT
#23	GGAATG CTG$_{82}$ (C)TGCTGGGGGGATCACAGACCAT
#11	GGAATG CTG$_{5}$ ----(Δ64 bp)----ATCGGGCGTTCGAC
#44	GGAATG CT----(Δ279 bp)----ACAGACCAT
#46	GGAATG CTG$_{5}$ C----(Δ260 bp)----ATCACAGACCAT
#17	CCTTGTAGCCGGGA----(Δ281 bp)----TCACAGACCATTTC
#19	TCCTTGTAGCCGGG----(Δ312 bp)----CTGAGGCCGACCTG
#31	TCGTCCTTGTAGCC----(Δ295 bp)----ATTTCTTTCTTTCG
#4 #7	(δ20)TTTGTCACTCATTATCCTATTACATTATCAATCCTT----(Δ3050 bp)----TCATTATCCTATTACATTATCAATCCTT GCATTTCAGCTTCC(δ18)
#39	(δ20)TAATATTAGGTATACAGAATATACTAG----(Δ2837 bp)----AGGTATACAGAATATACTAG AAGTTCTCCTCGAG(δ18)

***rad52*Δ junctions**

Parental allele	GGAATG CTG$_{80}$ CTGGGGGGATCACAGACCAT
#B10 #B12	GGAATG CTG CTGGGGGGATCACAGACCAT
#A3	GGAATG CTG$_{3}$ CTGGGGGGATCACAGACCAT
#C8	GGAATG CTG$_{4}$ CTGGGGGGATCACAGACCAT
#C5	GGAATG CTG$_{5}$ CTGGGGGGATCACAGACCAT
#H4	GGAATG CTG$_{8}$ CTG----(Δ262 bp)----CTGCAGCGAA
#A1	GGAATG C---------(Δ255 bp)-------------CCAT
#A2	GTCGTCCTTG----(Δ291 bp)---TGAGGCCGAC
#A11	AAGCTGGTCA----(Δ579 bp)---CGACCTGCAG
#B6	GGAATG----------(Δ252 bp)-------CAGACCAT
#B7	GGAATG CTG$_{3}$ CT-(Δ276 bp)-ACCTGCAGCG
#C9	GTCGTCCTTG----(Δ305 bp)---AGCGAAATCT
#C4	(δ20)ATCCCACCAATTATC-(Δ3021 bp)- CAATTATCAAAAAAT(δ18)
#C7	(δ20)TTATCTCAAAATTCAC-(Δ3029 bp)-AAAATTCACCTTTCTT (δ18)

***sae2*Δ junctions**

Normal allele	GGAATG CTG$_{77}$ CTGGGGGGATCACA
#A8 #D2 #D12	GGAATG CTG$_{3}$ CTGGGGGGATCACA
#D3	GGAATG CTG$_{72}$ (C)TGCTGGGGGGATCACA
#A2	GGAATG CTG$_{77}$ CGCTGGGGGGATCACA
#A4	GGAATG CTG$_{77}$ C(T)GGGGGGGGATCACA
#A5	GGAATG CTG$_{77}$ CCTGCTGGGGGGATCACA
#A6	GGAATG CTG$_{77}$ CTGCGCCTGGGGGGATCACA
#A11	GGAATG CTG$_{77}$ CTTGGTGGGGGGATCACA
#A12	GGAATG CTG$_{77}$ CGC(T)GGGGGGGATCACA
#B5	GGAATG CTG$_{77}$ CTGCTGCGCTGGGGGGATCACA
#E2	GGAATG CTG$_{18}$ CCTGCTGGGGGGATCACA
#B2	(δ20)TGTCACTCATTATCCTATTA -----(Δ3027 bp)-------TCATTATCCTATTACATTAT(δ18)

***dnl4Δ* junctions**

Parental allele	GGAATG CTG$_{80}$ CTGGGGGGATCACA
#A9	(δ20)AAGACTTAATATTAGGTATACAGAATATACTAGGAG----(Δ3029 bp)----CAGGTATACAGAATATACTAGAGTTCTCCTCGA(δ18)
#B2	(δ20)TTTCATTAGACTTTGTTACTGTT----(Δ3050 bp)----ACAGTTCCTCGTATCTTATGTCATCG(δ18)
#B7 #B9 #D3	(δ20)ATGTTTGGAAGAAAGGTGAATTTT----(Δ3052 bp)----GGTGAATTTTGAGATAATTGGTGGG(δ18)
#B12	(δ20)GTAAAGACTTAATATTAGGTATACAGAATATACTAG----(Δ3053 bp)----AGGTATACAGAATATACTAGAAGTTCTCCTCGAGGA(δ18)
#C10 #C12	(δ20)CTTATGTTATCTTCTTACACAGTAT----(Δ3049 bp)----ACACCGTATATGATAATATATTGGT(δ18)
#E8 #E9 etc.	(δ20)TGATACTTTGTCACTCATTATCCTATTACATTATCAA----(Δ3051 bp)----TCATTATCCTATTACATTATCAATCCTTGCATTTCAG(δ18)
#C9 #E11	(δ20)TGTTGGTAAAGACTTAATATTAGGTATACAGAATATACTAG----(Δ3054 bp)----TAATATCAGGTATACAGAATATACTAGAAGTTCTCCTCGAG (δ20)
#B5	(δ20)AAAGGTGAATTTTTTGATAATTG----(Δ2798 bp) +153 bp Tyδ19 chr.10----ATCCTATTACATTATCAATCCTT(δ19)
#D11	(δ20)TTGCTGGGATTCCATTGTTG----(Δ3147 bp) +94 bp Ty1δ21 chr.7----ATTACGATTATTCCTCATTC(δ18)

***sae2Δ dnl4Δ* junctions**

Parental allele	GGAATG CTG$_{81}$ CTGGGGGGATCACA
#B11	GGAATG CTG$_{81}$ CTGGGGGGATCACA
#B8 #C12 etc.	GGAATG CTG$_{80}$ CTGGGGGGATCACA
#C9 #D1 etc.	GGAATG CTG$_{79}$ CTGGGGGGATCACA
#A2	GGAATG CTG$_{3}$ CTGGGGGGATCACA
#A3	(δ20)GTTTCTCAATCCTTATGT----(Δ3050 bp)----CTTATGTCATCGTCTAACACCGTATATGA(δ18)
#A8	(δ20)GTTTCTCAATCCTTATGTTATC----(Δ3050 bp)----CTTATGTCATCGTCTAACACCGTAT(δ18)
#C4 #C5 etc.	(δ20)ATTACATTATCAATCCTTGCATTT----(Δ3051 bp)----CAATCCTTGCATTTCAGCTTCCTC(δ18)
#B6	(δ20)GTTTGGAAGAAAGGTGAATTTT----(Δ3052 bp)----AAAGGTGAATTTTGAGATAATT(δ18)
#B7 #C3	(δ20)ATATTAGGTATACAGAATATACTAG----(Δ3053 bp)----AGGTATACAGAATATACTAGAAGTT(δ18)
#A4	(δ20)GTATACAGAATATACTAG----(Δ3226 bp) +176 bp Tyδ12 chr.5----TCTAACACCGTATATGAT(δ19)
#C8	(δ20)TTCTTTGATA--(Δ25 bp) +23 bp Tyδ16 chr.10--TTATCAATCCTTGCATTT----(Δ3033 bp)----TTATCAATCCTTGCATTTCAGCTTCCTC(δ18)

***rad50Δ* junctions**

Normal allele	GGAATG CTG$_{80}$ CTGGGGGGATCACA
#A3	GGAATG CTG$_{<10}$ CTGGGGGGATCACA
#B2	GGAATG CTG$_{45}$ CTGGGGGGATCACA
#B4	GGAATG CTG$_{120}$ CTGCTGGGGGGATCACA
#B5	GGAATG CTG$_{115}$ CTGCTGGGGGGATCACA
#C1	GGAATG CTG$_{90}$ CTGGGGGGATCACA
#A2, #A4, #A6, #A7, #A8	(δ20)TCAATCCTTGCATTTTTAGC--------(Δ3050 bp)--------AGCTTCCTCTAACTTCGATG(δ18)
#A9, #A10, #A11, #A12	(δ20)AGAATATACTAGGAGTTCTC--------(Δ3053 bp)--------AAGTTCTCCTCGAGGATATA(δ18)
#B1, #B3, #B12	(δ20)AGAATATACTAGGAGTTCTC--------(Δ3053 bp)--------AGTTCTCCTCGAGGATATAG(δ18)
#B6, #B7, #B8, #B11	(δ20)ACTCATTATCCTATTACATTATCAA--------(Δ3029 bp)-------CATTATCCTATTACATTATCAATCC(δ18)
#B9	(δ20)TATACAGAATATACTAGGAGTTCTC--------(Δ3053 bp)-------AGAATATACTAGAAGTTCTCCTCGA(δ18)
#C2, #C3, #C4, #C5	(δ20)AGAATATACTAGGAGTTCTC--------(Δ3030 bp)--------AGTTCTCCTCGAGGATATAGG(δ18)
#C6	(δ20)TATCCTATTACATTATCAATCCTTGC--------(Δ3028 bp)--------TATTACATTATCAATCCTTGCATTTC(δ18)
#C8, #D2, #D3, #D5, #D7, #D9	(δ20)CTCATGTATCCTATTACATTATCAATCCTT--------(Δ3030 bp)--------TATCCTATTACATTATCAATCCTTGCATT(δ18)
#C9, #C10, #C11, #C12, #D6, #D8, #D10, #D11, #D12	(Tyδ4 chr.10)TCATAATTAATAAT--170 bp--ATTTTGAGATAATTG--ATTTTGAGATAATTGGTGGG (δ18)

Wild-type e*Sp*Cas9 junctions (guideRNA #1)

Parental allele	GGAATG CTG_{78} CTGGGGGGATCACAGACCATTTCTTT
C4#	TCGTCCTTGTAGCCGGG------(Δ280 bp)-----GGGGATCACAGACCATT
D12#	GGAATG CTG_{6}C--------(Δ2541 bp)--------GATGGAAATCATTATTGTAGT(δ18)
B4# B5#	(δ20)TATCTTCTTACACAGTAT------(Δ3050 bp)------GTATATGATAATATATTG(δ18)
A3# A5# etc.	(δ20)ACTTTGTCACTCATTATCCTATTACATTATCAA------(Δ3051 bp)------TCATTATCCTATTACATTATCAATCCTTGCATTT(δ18)
B1#	(δ20)TTCCATTGTTGGTAAAGACT(A)TAATAT------(Δ3053 bp)------ATTGTTGGTAAAGGCTATAATATCAGGTATACAGAAT(δ18)

Wild-type e*Sp*Cas9 junctions (guideRNA #2)

Parental allele	GGAATG CTG_{78} CTGGGGGGATCACAGACCATTTCTTT
#A8	GGAATG CTG_{17} TTG CTG_{5} CTGGGGGGATCACAGACCA
#A12	GGAATG CTG_{66} ----------G CTGGGGGGATCACAGACCA
#C4	GGAATG CTG_{25} (C)TTTGGGGGGATCACAGACCATTTCTTT
#A3	GGAATG CTG_{13} CTG--------(Δ238 bp)--------GATCACA
#A4	TAGCACCACGCC-----(Δ1342 bp)-----CCCCCGGGAGAT
#A6	TAATTTGTCAGT-----(Δ604 bp)------CTTGCAAGTTGA
#B6	TCTCGGTAGCCA------(Δ401 bp)------CGTTCGACTCGC
#B9	(δ20)ATAATATAATAGTAACATGAA------(Δ2404 bp)------TGTTGGAATAAAAATCAACTATCATC(δ19)
#A2 #A5 etc.	(δ20)TATCAATCCTTGCATTT-------(Δ3017 bp)-------TATCAATCCTTGCATTT(δ18)
#C9	(δ20)ATTGTTGGTAAAG-------(Δ3039 bp)-------ATTGTTGGTAAAG(δ18)
#E11	(δ20)ACTCATTATCCTATTACATTATCAATCCTTGCATTT------(Δ3051 bp)------TCCTTGCATTTCAGCTTCCTCTAAC(δ18)
#D10 #E3 #F7	(δ20)TTAATATTAGGTATACAGAATATACTAG------(Δ3053 bp)------AGGTATACAGAATATACTAGAAGTTCTCCTCGAG(δ18)
#F8	(δ20) TTCCTTCTTTGATA-------(Δ3112 bp)+59 bp Tyδ16 chr.10-------ATGACAGTTCCTCG(δ18)

Wild-type *Sp*Cas9 junctions (at the *ARG2* locus)

Parental allele	GGAATG CTG_{60} CTGGGGGGATCACAGACCATTTCTTT
#B10	GGAATG CTG_{72} CTGGGGGGATCACAGACCATTTCTTT
#A2 #A3 etc.	AGGCGCAAGACTTCAA----(Δ388 bp +TAGGGATAACAGGGTAAT)----CGAAATCTTGAGATCG
#B5	CATCTAGAGTCGTCCTTGTAGCC--(Δ325 bp)--TTTCTTTCTTTCGGCCAGGCTG
#A8 #B11	TCTCCACAGCAGCAGC-------(*YAK1-ARG2*)-------ATCACAGACCATTTCT

Supplemental Figure 1

Mosbach *et al.*
