# Supplementary figures and images for: Resection and repair of a Cas9 double-strand break at CTG trinucleotide repeats induces local and extensive chromosomal deletions
Source: PLoS Genet. 2020 Jul 16;16(7):e1008924. doi: 10.1371/journal.pgen.1008924 (PMC7413560; doi:10.1371/journal.pgen.1008924)

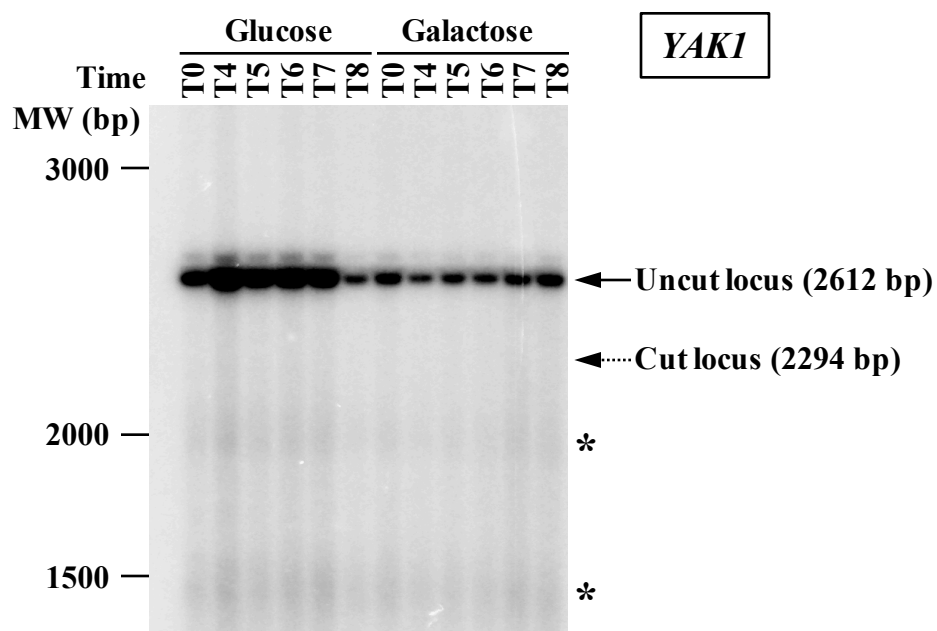

Supplemental Figure 2  
Mosbach et al.

Supplement: S2 Fig — The time course was run in non-induced (glucose) and induced (galactose) conditions, as previously. The uncut locus is clearly visible as a 2612 bp band, but no signal can be seen at the expected size for a DSB (2294 bp). Two fuzzy bands present in both conditions and corresponding to faint cross-hybridizations are indicated by asterisks. Note that even when the blot was overexposed no signal could be detected at the expected DSB size. (PDF) [file pgen.1008924.s002.pdf]

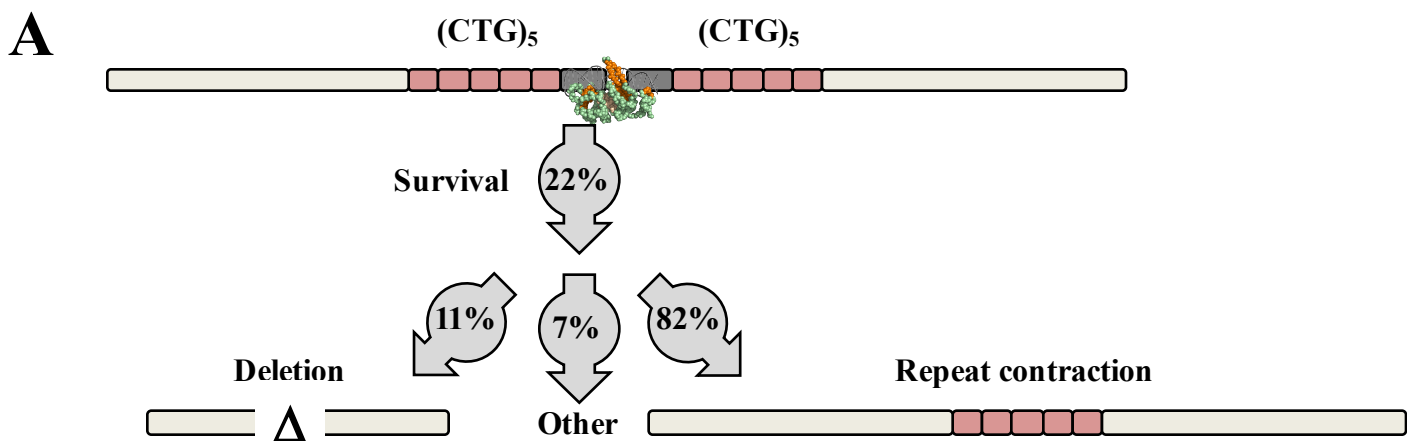

*Richard et al., 1999, Mol. Gen. Genet.*

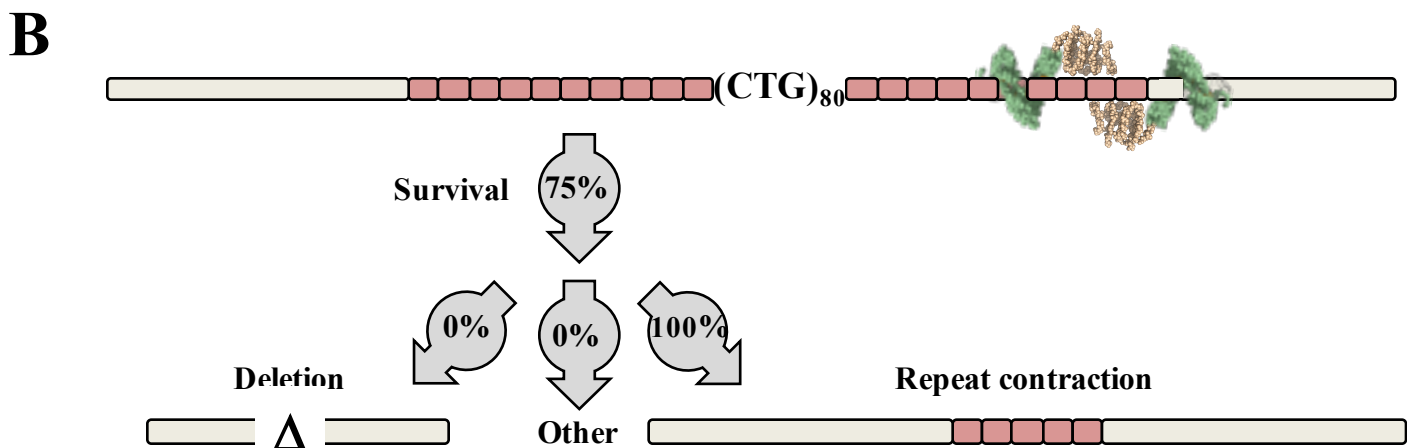

*Richard et al., 2014, PLoS ONE, Mosbach et al., 2018, Cell Reports*

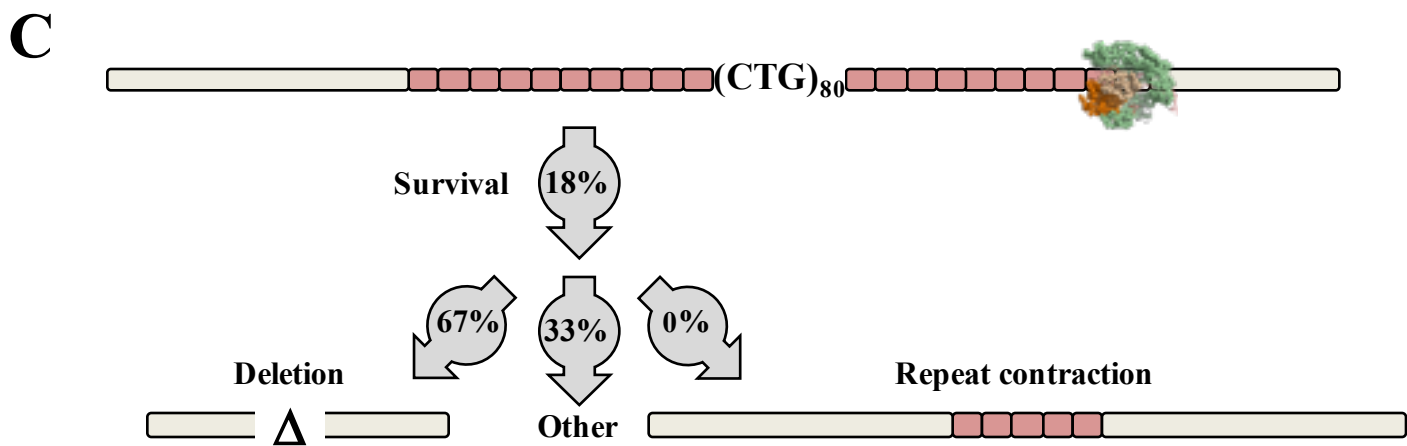

Supplement: S4 Fig — The DSB induced by I-SceI (A), a TALEN (B) or SpCas9 (C). In each case, survivors were separated in three different categories: deletions (local or large) around the repeat tract, removing partially or totally the repeat (left), repeat contraction without other mutation (right) or all other kinds of rearrangements (middle). Note that in A, five triplets flank the repeat tract on each side, whereas in B and C the break is made at the end of a long (80 triplets) repeat tract, leaving 1–4 triplets downstream and the remaining upstream the DSB. (PDF) [file pgen.1008924.s004.pdf]

A

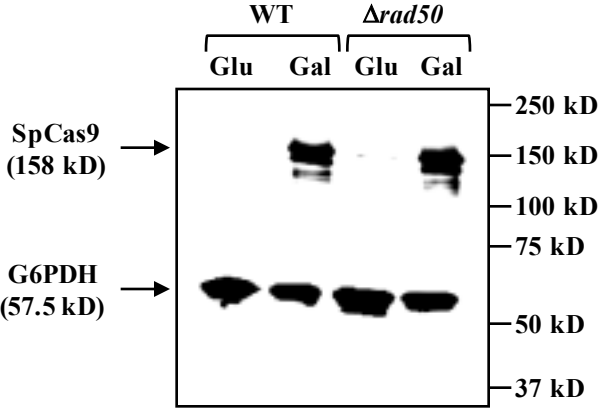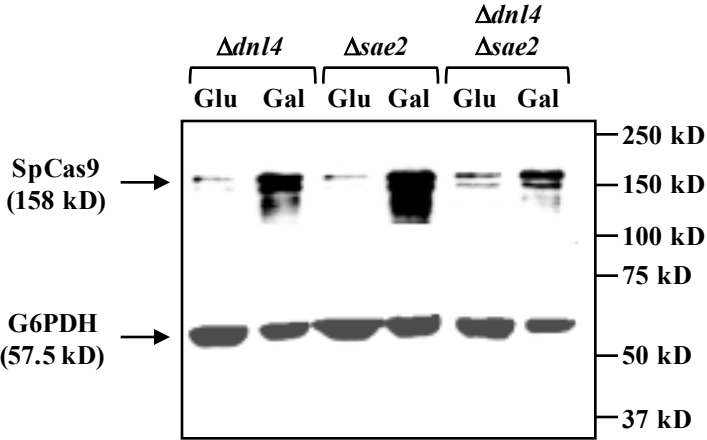

B

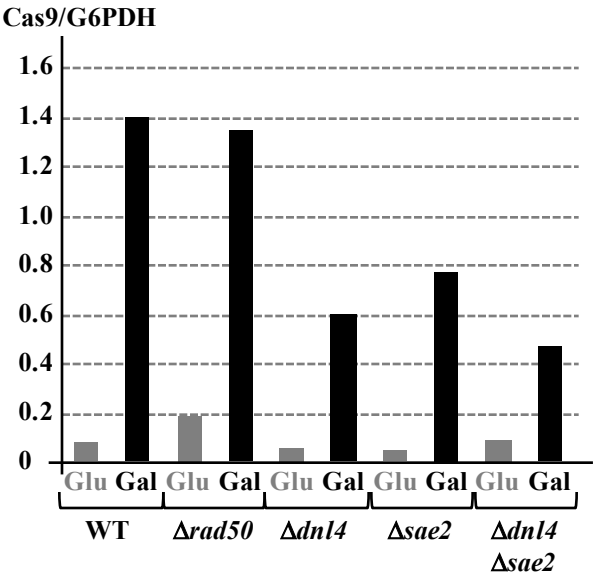

Supplement: S5 Fig — A: Western blots. Proteins were extracted in non-induced (Glu) and induced (Gal) conditions for wild type and each mutant strain. Glucose-6-phosphate deshydrogenase (G6PDH) was used as a loading control. B: Ratios of Cas9 over G6PDH signals, for each strain in each condition. (PDF) [file pgen.1008924.s005.pdf]
