# Supplementary material for: Resection and repair of a Cas9 double-strand break at CTG trinucleotide repeats induces local and extensive chromosomal deletions
Source: PLoS Genet. 2020 Jul 16;16(7):e1008924. doi: 10.1371/journal.pgen.1008924 (PMC7413560; doi:10.1371/journal.pgen.1008924)
Supplement: S3 Fig — A: Real-time PCR quantification of CDC8 and JEM1 amounts relative to an internal control on chromosome IV, in diploid cells in which Cas9 was induced. Half the amount of CDC8 product was detected in each clone analyzed. This was significantly different from the amount of product amplified from the JEM1 gene located on the other chromosome X arm. B: Illumina results for diploid and haploid cells. For each clone, the number of mutations detected is shown. Substit.: nucleotide substitution; Indel: insertion or deletion; Indel micro.: insertion or deletion of one repeat unit in a microsatellite. The asterisk corresponds to a 36 bp deletion in the FLO11 minisatellite (36 bp repeat). (PDF) [file pgen.1008924.s003.pdf]

A

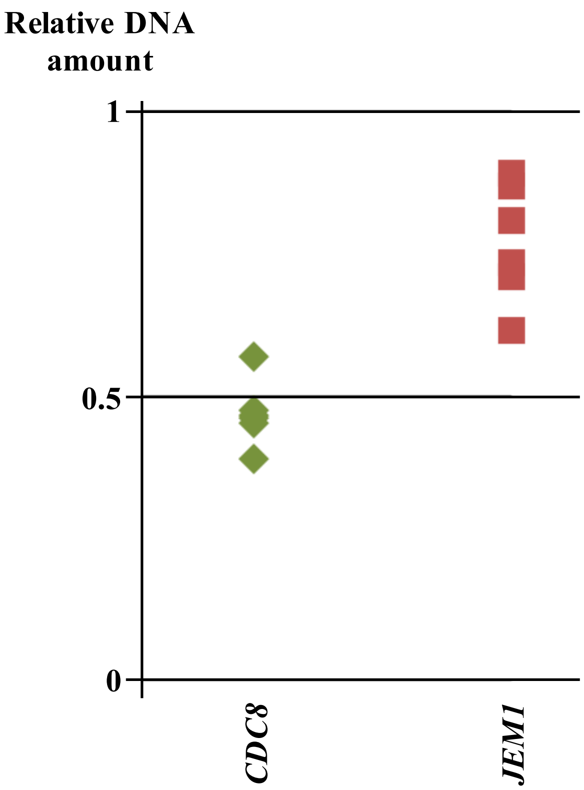

B

Diploids: Cas9 repressed Clone	Substit.	Indel	Indel micro.	Diploids: Cas9 expressed Clone	Substit.	Indel	Indel micro.	Haploids: Cas9 expressed Clone	Substit.	Indel	Indel micro.
1	-	-	-	1	-	-	1	1	-	-	-
2	1	-	-	2	-	-	1	2	1	-	-
3	1	-	-	3	-	-	-	3	-	-	-
4	-	-	-	4	-	-	-	4	-	-	-
5	-	-	-	5	-	-	-	5	-	-	-
**Total**	**2**	**0**	**0**	6	-	-	-	6	1	-	-
				7	-	-	-	7	1	-	-
				8	-	-	-	8	2	-	-
				9	-	-	-	9	2	-	-
				10	-	-	-	10	1	-	-
				11	-	-	-	**Total**	**8**	**0**	**0**
				12	-	-	-				
				13	-	-	-				
				14	-	-	-				
				15	1	-	-				
				16	-	1*	-				
				17	-	-	-				
				18	2	-	-				
				**Total**	**3**	**1**	**2**				

Supplemental Figure 3
Mosbach *et al.*
